# Sanhuang Xiexin decoction promotes good functional outcome in acute ischemic stroke

**DOI:** 10.1002/brb3.1185

**Published:** 2018-12-19

**Authors:** Juexian Song, Xin Chen, Yi Lyu, Wei Zhuang, Jing Zhang, Li Gao, Xiaolin Tong

**Affiliations:** ^1^ Department of Neurology Xuanwu Hospital, Capital Medical University Beijing China; ^2^ Department of Geriatrics Shanghai East Hospital, Tongji University School of Medicine Shanghai China; ^3^ Department of Anesthesiology Yunnan Baoshan Anli Hospital Shanghai China; ^4^ Department of Pharmacy Xuanwu Hospital, Capital Medical University Beijing China; ^5^ Department of Endocrinology Guang’anmen Hospital of China, Academy of Chinese Medical Sciences Beijing China

**Keywords:** acute ischemic stroke, endovascular intervention, inflammatory factors, Sanhuang Xiexin decoction

## Abstract

**Objectives:**

To explore the efficiency and safety of Sanhuang Xiexin decoction in the treatment of acute ischemic stroke (AIS) patients after endovascular intervention examination.

**Methods:**

In this prospective observational study, 121 AIS patients admitted in our hospital were enrolled from January 2012 to December 2015. They were randomly divided into two groups, 61 patients received Sanhuang Xiexin decoction + basic treatment (SX group) and 60 patients received basic treatment (control group). The prescription of Sanhuang Xiexin decoction was taken in the SX group, with one dose (100 ml), twice a day for 7 days orally. For all patients, blood samples were drawn on the first morning and sixth morning after endovascular intervention examination under fasting state for Fib (fibrinogen), PAgT (platelet aggregation test), CRP (C‐reactive protein), and TMAO (trimethylamine oxide) tested. Estimate the changes in plasma Fib, PAgT, CRP, and TMAO levels and the syndrome of fire‐heat scores.

**Results:**

The plasma Fib, PAgT, CRP, and TMAO levels in the SX group were significantly lower than those in the control group (P_Fib_ < 0.01, P_PAgT_ < 0.01, P_CRP_ = 0.02, P_TMAO_ < 0.01). The syndrome of fire‐heat scores in the SX group was significantly lower than that in the control group (*p* < 0.01). The incidences of ischemic cerebrovascular events within 3 and 6 months after endovascular intervention treatment in the SX group were lower than those in the control group (P_3 month_ = 0.04, P_6month_ = 0.03).

**Conclusions:**

The prescription of Sanhuang Xiexin is efficient and safe in the treatment of AIS patients after endovascular intervention examination through reducing the inflammatory factors.

## 1 INTRODUCTION

Stroke is one of the most common causes of death and disability worldwide (Li, Geng, & Ding, 2017). The treatment of acute ischemic stroke (AIS) is a very complicated process, but the effect of Western medicine treatment is not satisfactory (Xu & Li, 2011). The traditional Chinese medicine has been used in vascular disease treatment for thousands of years in the Asia‐Pacific region. It has been proven that the traditional Chinese medicine can play the advantage of treating both root causes and symptoms of inflammatory response in AIS patients with endovascular intervention examination (Zhang et al., 2015).

According to the theory of traditional Chinese medicine, AIS patients with syndromes of fire‐heat, such as dry mouth, constipation, yellow urine, and red tongue, should receive clearing heat and purging phlegm treatment (Zou, 1999), so we chose the prescription of Sanhuang Xiexin decoction, which included Coptidis Rhizoma, Rhei Radix et Rhizoma, Scutellariae Radix, Lophatherum Gracile, Bile Arisaema, and Forsythia (Chen, 2008). Sanhuang Xiexin decoction has been demonstrated to have a variety of bioactivities including anti‐inflammation (Shih, Chen, Wu, & Lo, 2011), anti‐oxidative (Liou, Hsu, Liang, & Yeh, 2012), and anti‐atherosclerosis (Wang, Lin, Cheng, & Juo, 2011). In this prospective, randomized, and controlled study, we explored the efficiency and safety of Sanhuang Xiexin decoction in the treatment of AIS patients after endovascular intervention examination.

## 2 MATERIALS AND METHODS

### 2.1 Participants

From January 2012 to December 2015, 121 patients with AIS who were admitted in the neurology department of our hospital were enrolled in this study. This study was approved by the local ethical committee, and all patients signed informed consents before treatment. They were in accordance with the diagnostic criteria of cerebral infarction approved by the fourth national cerebrovascular academic conference (1995).

Inclusion and exclusion criteria: (1) All enrolled patients underwent endovascular intervention examination (multislice computed tomography angiographic, MSCTA; parameters: 120 kV, 150 mAs, thickness 5 mm, interval 5 mm); (2) all patients met the standards of fire‐heat scores (a. tongue: 5 points for red tongue, 6 points for deep red tongue; b. tongue coating: 2 points for thin‐yellow coating, 3 points for thick‐yellow coating, 4 points for dry coating, 5 points for gray‐black‐dry coating; c. excrement: 3 points for astriction within 3 days, 4 points for astriction more than 3 days; d. expression: 2 points for irritability, 3 points for agitated and restless, 4 points for delirium; e. face breath odor: 2 points for loud, rough, or dry‐red lips, 3 points for flame, panting, or halitosis; f. fever: 3 points for fever; g: pulse: 2 points for powerful pulse; g. mouth feeling: 1 point for bitter taste and dry throat, 2 points for thirst with a predilection for cold drink; h. urine: 1 point for odynuria; Table 1); (3) patients without incomplete hepatic or renal function or severe psychotic disease; (4) patients without high sensitivity to medicine in this study; (5) all patients were informed by both the experts in the interventional department and the experts in neurology department, and the family members were informed of the disease and signed the informed consent to be examined by cerebral angiography and to be given the decoction of Sanhuang Xiexin.

**Table 1:** Standards of fire‐heat scores

Items	Symptoms	Scores
Tongue	Red tongue	5
	Deep red tongue	6
Tongue coating	Thin‐yellow coating	2
	Thick‐yellow coating	3
	Dry coating	4
	Gray‐black‐dry coating	5
Excrement	Astriction within 3 days	3
	Astriction more than 3 days	4
Expression	Irritability	2
	Agitated and restless	3
	Delirium	4
Face breath odor	Loud, rough, or dry‐red lips	2
	Flame, panting, or halitosis	3
Fever	Fever	3
Pulse	Rough pulse	2
Mouth feeling	Bitter taste and dry throat	1
	Thirst with a predilection for cold drink	2
Urine	Odynuria	1

### 2.2 Therapeutic methods

All patients who were enrolled in this study were randomly divided into Sanhuang Xiexin decoction group (SX group, *n* = 61) and control group (*n* = 60). Basic treatment with drugs for antiplatelet and improving circulation was used in the control group (aspirin 300 mg and clopidogrel 75 mg were used pre‐endovascular intervention treatment with once a day for 3–5 days; the same dose was used post‐endovascular intervention therapy; aspirin 300 mg reduced to 100 mg 6 weeks after endovascular intervention therapy). In the SX group, the prescription of Sanhuang Xiexin decoction (Coptidis Rhizoma, Rhei Radix et Rhizoma, Lophatherum Gracile, Bile Arisaema, and Forsythia (2:1:2:2:2) were thoroughly decocted in water for 2 hr (1:15, w/v), then the gruffs were extracted three times using boiling water (1:10, w/v), for another 2 hr, filtered by gauze. Finally, all the herbs were merged and concentrated to 1.0 g/ml; Wei, Tao, Cui, Jiang, Qian, & Duan, 2017; Zhang, Wang, Ma, Zhu, & Wang, 2013) was taken on the basis of basic treatment, with one dose (100 ml), twice a day for 7 days orally.

### 2.3 Study design

According to the criteria of diagnosis and curative effect evaluation of stroke (Trial) (State Administration of Traditional Chinese Medicine, 1996), all patients were evaluated by the same group of trained neurologists with the standard of syndrome fire‐heat scores (Wang, Han, & Chen, 1996).

The blood samples were drawn on the first morning and the sixth morning after endovascular intervention therapy under fasting state for Fib (Fibrinogen), PAgT (platelet aggregation test), CRP (C‐reactive protein), and TMAO (trimethylamine oxide) tested. Observing the changes in plasma Fib, PAgT, CRP, and TMAO levels and the syndrome of fire‐heat scores.

The patients were followed up for 6 months after the endovascular intervention treatment to observe the occurrence of cerebrovascular events within 3 and 6 months, including transient ischemic attack, acute ischemic stroke, and vascular restenosis.

### 2.4 Statistical analyses

Statistical analyses were performed using SPSS 17.0 software (IBM SPSS, Armonk, NY, USA). The continuous variables fitting the normal distribution were expressed as mean ± standard deviation (*SD*). The Student's *t* test was used for continuous variables, and the chi‐squared test was used for categorical variables. *p* < 0.05 was considered to be statistically significant.

## 3 RESULTS

### 3.1 Comparison of plasma Fib, PAgT, CRP, and TMAO levels and syndrome of fire‐heat scores in the first morning and sixth morning after endovascular intervention treatment

The two groups were comparable with no significant difference in gender, age, and complications. There were no significant differences between the SX group and the control group in the plasma levels of Fib, PAgT, CRP, and TMAO and syndrome of fire‐heat scores in the first morning after endovascular intervention treatment (*p* > 0.05; Table 2). The plasma Fib, PAgT, CRP, and TMAO levels in the SX group were significantly lower than those in the control group in the sixth morning after endovascular intervention treatment (P_Fib_ < 0.01, P_PAgT_ < 0.01, P_CRP_ = 0.03, P_TMAO_ < 0.01). The syndrome of fire‐heat scores in the SX group was also lower than that in the control group (*p* < 0.01; Table 3).

**Table 2:** The serum levels of Fib, PAgT, CRP, and TMAO and fire‐heat scores in the first morning after endovascular intervention treatment ($\bar{x}$ ± s, *n *[%])

	SX group (*n* = 61)	Control group (*n* = 60)	*p* Value
Age	59.15 ± 13.76	60.78 ± 12.13	0.78
Male	33 (55.00)	32 (53.33)	0.86
Hypertension	28 (45.90)	29 (48.33)	0.79
Diabetes	14 (23.33)	13 (21.31)	0.87
Heart disease	9 (14.75)	9 (15.00)	0.97
Smoking	32 (52.46)	30 (50.00)	0.79
Drinking	18 (29.51)	15 (25.00)	0.58
Fib	4.20 ± 0.90	4.15 ± 0.61	0.74
PAgT	47.60 ± 7.28	45.73 ± 6.22	0.67
CRP	5.60 ± 2.47	5.53 ± 2.84	0.62
TMAO	276.58 ± 48.59	287.82 ± 52.18	0.53
Syndrome of fire‐heat scores	13.20 ± 6.50	12.04 ± 6.973	0.69

Note

The clinical data of the two groups were compared, the *p* values were >0.05, and there were no statistical differences.

CRP: C‐reactive protein; Fib: fibrinogen; PAgT: platelet aggregation test; SX: Sanhuang Xiexin decoction; TMAO: trimethylamine oxide.

**Table 3:** The serum levels of Fib, PAgT, CRP, and TMAO and fire‐heat scores in the sixth morning after endovascular intervention treatment,$\bar{x}$ ± s

	SX group (*n* = 61)	Control group (*n* = 60)	*p* Value
Fib	2.62 ± 0.97	3.72 ± 0.91	<0.01
PAgT	33.49 ± 14.70	44.53 ± 14.71	<0.01
CRP	3.22 ± 2.10	4.38 ± 2.14	0.03
TMAO	145.04 ± 32.12	231.58 ± 47.43	<0.01
Syndrome of fire‐heat scores	6.84 ± 3.60	11.16 ± 2.85	<0.01

Note

CRP: C‐reactive protein; Fib: fibrinogen; PAgT: platelet aggregation test; SX: Sanhuang Xiexin decoction; TMAO: trimethylamine oxide.

### 3.2 Comparison of the incidence of cerebrovascular events within 3 months and 6 months after endovascular intervention treatment

The total incidence of cerebrovascular events (transient ischemic stroke, acute ischemic stroke, and vascular restenosis) in the SX group was lower than that in the control group within 3 and 6 months after endovascular intervention treatment (P_3month_ = 0.01, P_6month_ = 0.03; Table 4 **)**.

**Table 4:** Cerebrovascular events within 3 and 6 months after endovascular intervention treatment, *n* (%)

	SX group (*n* = 61)	Control group (*n* = 60)	*p* Value
Cerebrovascular events within 3 months	2 (3.28)	10 (16.67）	0.01
Cerebrovascular events within 6 months	6 (9.84)	16 (26.67）	0.03

Note

SX: Sanhuang Xiexin decoction; cerebrovascular events: transient ischemic stroke, acute ischemic stroke, and vascular restenosis.

## 4 DISCUSSION

In this study, we found (a) Sanhuang Xiexin decoction could significantly decrease the levels of inflammatory factors including Fib, PAgT, CRP, and TMAO in ischemic stroke patients; (b) in addition, Sanhuang Xiexin decoction could also reduce the syndrome of fire‐heat scores; (c) moreover, Sanhuang Xiexin decoction could reduce the incidence of ischemic cerebrovascular events in ischemic stroke patients within 3 and 6 months after endovascular intervention treatment as well.

Inflammation plays an important role in the process of ischemic stroke, and inflammatory factors are hallmarks of inflammation. After the onset of acute ischemic stroke, the inflammatory activities could further promote the brain damage, and C‐reactive protein is a common inflammatory factor which has been reported that its level is connected with the severity of neurological deficit and disability after stroke (Das, Roy, Kaul, Jyothy, & Munshi, 2004). In addition, fibrinogen, which was also detected in this study, is regarded as a key regulator of inflammation in disease. The cellular mechanisms for fibrinogen functions in different tissues have been identified, and the role of fibrinogen is not only a marker of vascular rapture but also a multi‐faceted signaling molecule which can enhance the protection from infection and extensive inflammation (Davalos & Akassoglou, 2012). More importantly, previous study showed that the fibrinogen knockout mice had a significantly improved cerebral reperfusion in the absence of fibrinogen deposition and a marked reduction in brain infarction after ischemia–hypoxia (Zacharowski et al., 2007). Previous studies have reported that platelet aggregation test was higher in ischemic stroke patients compared to healthy people (Schmalbach et al., 2015) and the high level of platelet aggregation test might contribute to the poor outcome of ischemic stroke (Choi, Cha, & Huh, 2014). Some studies have shown that the incidence of AIS is related to intestinal microflora. Intestinal microbiota and its metabolite TMAO/LPS can enhance platelet aggregation, increase inflammatory response, and influence the occurrence and prognosis of AIS (Zhu, Gregory, Org, Buffa, & Hazen, 2016). The prescription of Sanhuang Xiexin decoction has the functions of clearing away heat and toxic materials, resolving phlegm and dredging collaterals, clearing bowels and purging diarrhea, regulating intestinal flora through purgation, reducing the production of TMAO, reducing platelet aggregation and inflammatory reaction, and improving stroke and prognosis. This study showed that the Sanhuang Xiexin decoction could effectively reduce the blood levels of Fib, CRP, PAgT, and TMAO and the fire‐heat scores of AIS patients when compared with the control group, which proved the clinical efficacy of this traditional Chinese medicine prescription.

Sanhuang Xiexin decoction was first described in "On Cold Damage (Shang Han Lun)", a famous traditional Chinese medicine book, and the preparation of it was also recorded in detail (Li, 1997). Coptidis Rhizoma (the Ranunculaceae family, rhizomes of Coptis Chinensis Franch), Rhei Radix et Rhizoma (the Polygonaceae family, rhizomes of Rheum officinale Baill), and Scutellariae Radix (the Labiatae family, roots of Scutellaria baicalensis Georgi) are the most important components of it. Palmatine, berberine, jatrorrhizine, and coptisine are the main functional components in Coptidis Rhizoma which have many pharmacological effects including anti‐inflammatory, anti‐diabetic, antioxidative, and hypercholesterolemic effects (Yuan, Tu, Ye, & Wu, 2006). Anthraquinones which contain emodin, rhein, and aloe‐emodin are the main active constituents of Rhei Radix et Rhizoma. They not only have antimicrobial and antiviral properties but also can increase motor locomotion of the large intestine (Semple, Pyke, & Reynolds, 2011). The indicators of quality control for Scutellariae Radix are usually baicalin, wogonosideas, and wogonin in view of their various pharmacological effects of anti‐inflammatory, antibacterial, anti‐arthritic, antitumor, antioxidant activities, and free radical scavenging (Gao, Huang, Yang, & Xu, 1999). There have been a number of studies to detect the effects of Sanhuang Xiexin decoction on inflammation. In animal studies, the results indicated that the decoction of Sanhuang Xiexin could significantly increase IL‐10 production as well as decrease TNF‐α production which demonstrated that the Sanhuang Xiexin decoction had significant anti‐inflammatory effects (Zhang, Ma, Wang, & Wang, 2013). Lophatherum Gracile contains triterpenes and steroids, including arundoin, imperata, taraxerol, beta‐sitosterol, and campesterol. It has antipyretic, diuretic, and antibacterial effects (Chen, 2014). Lophatherum Gracile is also rich in flavonoids, including orientin, isoorientin, and vitexin; it can inhibit the expression of NO and iNOS which is mediated by LPS in macrophages; and it also can significantly reduce the expression of TNF, IL‐1, and IL‐6 (Yang et al., 2017). Bile Arisaema can remove heat‐phlegm which is effective for stroke (Bai et al., 2010). It can inhibit the production of pro‐inflammatory cytokines, including IL‐6 and tumor necrosis factor (TNF); moreover, it also can inhibit the expression of mRNA and protein during the production of cytokine (Ahn, 2012). Forsythia has the liver protective, antipyretic, anti‐inflammatory, and diuretic effects (Yuan et al., 2017).

In summary, this prospective randomized controlled clinical study showed that Sanhuang Xiexin decoction was effective and safe in the treatment of acute cerebrovascular disease, and it could relieve constipation, thick‐yellow tongue coating, and other clinical symptoms. It could also reduce the inflammatory factors after AIS. Besides, this traditional Chinese medicine prescription had an inhibitory effect on the inflammatory activity after endovascular intervention examination in patients with AIS without side effects. However, we did not record the cerebral angiographic data, and we could not compare the efficacy between different types of AIS. In further investigation, we should continue to improve the composition in this prescription to get better curative effect, and the mechanism of this prescription should also be explored.

## CONFLICT OF INTEREST

None declared.
